# The Differential Effects of *Tuta absoluta* Infestations on the Physiological Processes and Growth of Tomato, Potato, and Eggplant

**DOI:** 10.3390/insects13080754

**Published:** 2022-08-22

**Authors:** Lindiwe Mahlangu, Phumzile Sibisi, Robert S. Nofemela, Titus Ngmenzuma, Khayalethu Ntushelo

**Affiliations:** 1 Department of Agriculture and Animal Health, University of South Africa, Private Bag X6, Florida 1710, South Africa; 2 Agricultural Research Council–Plant Health and Protection, Private Bag X134, Queenswood 0121, South Africa; 3 Department of Chemistry, Tshwane University of Technology, Private Bag X680, Pretoria 0001, South Africa

**Keywords:** eggplant, leaf gas exchange, herbivory, tomato pinworm, physiological functioning

## Abstract

**Simple Summary:**

Our study aimed to assess how *Tuta absoluta* affects different Solanaceae plant species. It was observed that susceptible plants experience diminished growth and reproduction rates, which may be a result of a physiological malfunction. Infestation by *T. absoluta* caused 100% crop loss to tomato plants, whereas significant yield reduction was observed for potato plants, and no significant feeding effect was observed for eggplant. The affected plants also experienced physiological impairments. This study is essential in that it demonstrates the differential risk of *T. absoluta* infestations on the production of the tested Solanaceae plant species.

**Abstract:**

*Tuta absoluta* (Meyrick, 1917) (*Lepidoptera*: *Gelechiidae*) is a destructive insect pest toward crops and belongs to the Solanaceae family. Since it was first recorded in South Africa in 2016, the pest has spread extensively and caused tremendous damage to field and tunnel-grown tomato crops. This study aimed to investigate how *T. absoluta* affects the growth and physiology of three Solanaceae plant species: tomato, potato, and eggplant. These three crops were infested with L1 instar larvae, and their growth and physiology were assessed during insect feeding. The damage to the infested tomato host plant was severe, with *T. absoluta* destroying 100% of the plants. The tomato plants were distorted 15 days after infestation, that is, before the fruit set. For potato, the defoliation was moderate, but the infested plants produced fewer tubers compared to the uninfested host plants. Eggplant had fewer visible signs of feeding, resulting in no significant difference between the infested and uninfested host plants in terms of growth and physiological functions. Infested tomato and potato plants had stagnant growth, fewer and damaged leaves, a reduced chlorophyll content, a reduced photosynthesis rate, a poor transpiration rate, poor water conductance, and poor intercellular carbon dioxide concentrations. This study closes the knowledge gap on the morphological (growth) and physiological responses of different Solanaceae species to *T. absoluta* infestation, and it also demonstrates the differential risk of *T. absoluta* infestations in the production of tomato, potato, and eggplant.

## 1. Introduction

Aggressive exotic insect pests are able to adapt well when introduced into a new area and cause considerable damage to high-value crops [1]. Their introduction often reduces crop yield significantly, which compels farmers to invest more in pest control strategies [2]. Most invasive insect pests belong to the order Lepidoptera [3]. In 2016, *Tuta absoluta* (Meyrick, 1917) (*Lepidoptera*: *Gelechiidae*) invaded South Africa [4], with this now causing significant yield losses to both open fields and tunnel-grown tomatoes across the country. *Tuta absoluta* has also spread fast through different regions of the world since its accidental introduction into Spain in 2006, and it is now considered the greatest threat to global tomato production [5]. The larvae feed on tomato leaves, stems, and fruits at any stage of a plant’s development, leading to reduced yield and fruit quality [6,7]. When no pest control measures are implemented or when they are not effective, the damage caused by *T. absoluta* can cause 100% crop loss [5]. Although tomato (*Solanum lycopersicum* L.) is the preferred host plant, *T. absoluta* has been reported to feed on the foliage of other Solanaceae plant species of which, potato (*Solanum tuberosum*) and eggplant (*Solanum melongena*) are their major alternative host plants, also feeding on 10 other Solanaceae species [8,9,10]. However, the effects of genetic variation on how different populations of *T. absoluta* utilize Solanaceae host plant species has been reported [11], and it is imperative to study its relationship with Solanaceae crops in different regions.

Potato is the most produced Solanaceae crop in South Africa at 2,491,000 tons, followed by tomato at 604,000 tons during the 2019/20 season, whereas eggplant is not a major crop [12]. As most of South Africa’s landscape is suitable for the normal development of *T. absoluta* [13], it is imperative to understand the effects of its infestations on the production of these crops. Studies on the range of *T. absoluta* host plants have only considered the effects of different Solanaceae species on oviposition, feeding preferences, and progeny fitness [14,15,16]. However, it is also important to understand how *T. absoluta* infestations affect the growth and development (physiological function) of the different Solanaceae species. The limited available information on tomato has only considered plant growth parameters, and little has been regarded in terms of physiological parameters [17], with no information on potato and eggplant.

To close the knowledge gap, this study aimed to perform a comparative investigation between infested and uninfested tomato, potato, and eggplant host plants. This was conducted to determine the growth rate and physiological responses of tomato, potato, and eggplant host plants to *T. absoluta* infestation and to further assess the correlation between growth and physiological parameters. When considered in their entirety, the objectives of this study are essential for evaluating the risk that *T. absoluta* poses to the production of these important Solanaceae crops.

## 2. Materials and Methods

### 2.1. Experimental Site

The study was conducted at the Science Campus in Florida, South Africa, of the University of South Africa, between March and August 2021. Experimental plants were maintained in the greenhouse under a 28 °C (day)–8 °C (night) temperature regime with overhead irrigation.

### 2.2. Research Design

#### 2.2.1. Host Plants

Tomato (cv Heinz), potato (cv Valor), and eggplant (cv Black Beauty) were used for this study. The seeds were directly cultivated in 25 cm experiential pots, which contained potting soil. After the development of the first true leaves, the plants were fertilized with nitrosol to promote healthy leaf development. One cap of nitrosol foliar fertilizer (Efekto Pty [Ltd.], Benmore, South Africa) was diluted in 3 L of water and sprayed onto the leaves. This was repeated at fortnightly intervals. The experimental tomato plants used were at BBCH (Biologische Bundesanstalt, Bundessortenamt, and chemical industry) growth stage 17. The potato plants were at growth stage 17, and the eggplants were at growth stage 16. Ten plants for each species were used for the experiments [18].

#### 2.2.2. Insect Infestation

**Figure 1:**
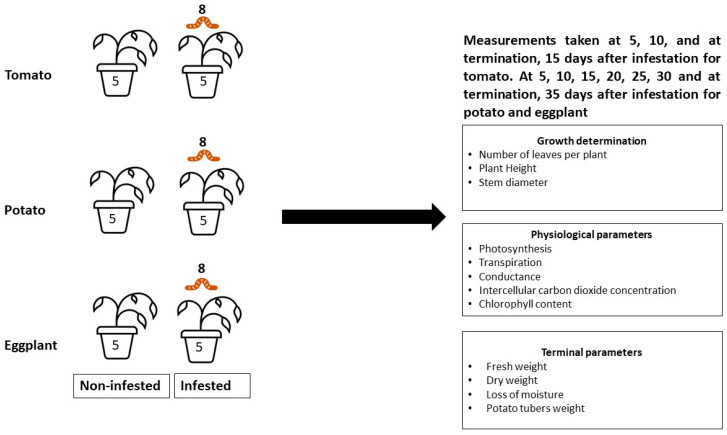
Experimental setup to determine the effects of *Tuta absoluta* infestations on the growth and physiological parameters of tomato, potato, and eggplant.

The *Tuta absoluta* larvae, used to infest the plants, were taken from a stock culture at the Agricultural Research Council-Plant Health and Protection, Roodeplaat Campus, Pretoria, South Africa. Five plants from each crop were infested with eight L1 *T. absoluta* instar larvae, and the other five plants remained uninfested (Figure 1). The infested plants were individually covered with net cages and placed randomly on wire mesh tables.

### 2.3. Data Collection

#### 2.3.1. Plant Morphological (Growth) Parameters

**Figure 2:**
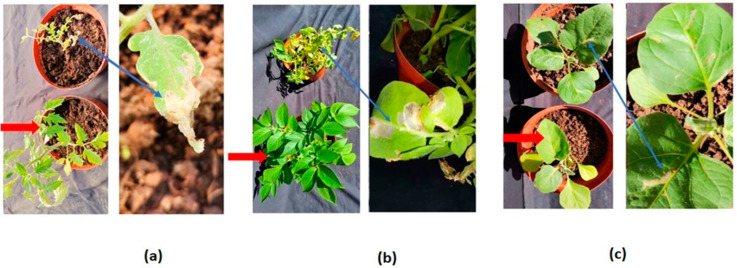
Tomato (**a**), potato (**b**), and eggplant (**c**) infested with *Tuta absoluta* (indicated with a blue arrow) and uninfested plants (indicated with a red arrow).

The number of leaves per plant, along with the plant height, stem diameter, and tubers or fruits per plant, were recorded before infestation and at five-day intervals for both the infested and uninfested plants. The feeding damage of *T. absoluta* on the host plants is shown in Figure 2.

#### 2.3.2. Plant Physiological Parameters

The chlorophyll content, recorded every five days, was measured using a CCM-200 plus GPS chlorophyll content meter. The photosynthesis rate, transpiration rate, water conductance, and intercellular carbon dioxide were measured using a portable circuit, infrared gas analysis system (Li-6400, Li-Cor Inc., Lincoln, NE, USA). The Li-6400 measures photosynthesis in real-time using an infrared gas analyzer, which is placed in the leaf chamber. Gas exchange measurements were captured during the daytime under a clear sky between 09:59:28 and 10:12:31. The tomato host plants were terminated at senescence, 15 days after infestation, while the potato and eggplant host plants were terminated 35 days after infestation.

### 2.4. Fresh Weight, Dry Weight, and Loss of Moisture

At termination, the plants were uprooted and immediately weighed to determine their fresh weight. Subsequently, the plants were kept in brown bags in an oven at 67 °C for 48 h, and then weighted to obtain their dry weight. To determine the loss of moisture between the fresh weight and the dry mass, the dry mass was subtracted from the fresh weight.

### 2.5. Statistical Analysis

Data were computed statistically with IBM SPSS statistics 27. Data on growth and physiological parameters were analyzed using a two-tailed and unpaired *t*-test. *p* < 0.05 was used to determine the significance of results between the infested and uninfested host plants. Data on the different time points were analyzed individually and were interpreted as such to respond to the research question: how do the three crops respond morphologically and physiologically to *T. absoluta* infestation? Pearson correlation coefficients were calculated to assess the relationship between the measured growth and physiological parameters.

## 3. Results

### 3.1. Growth Parameters

#### 3.1.1. Number of Leaves Per Plant

**Figure 3:**
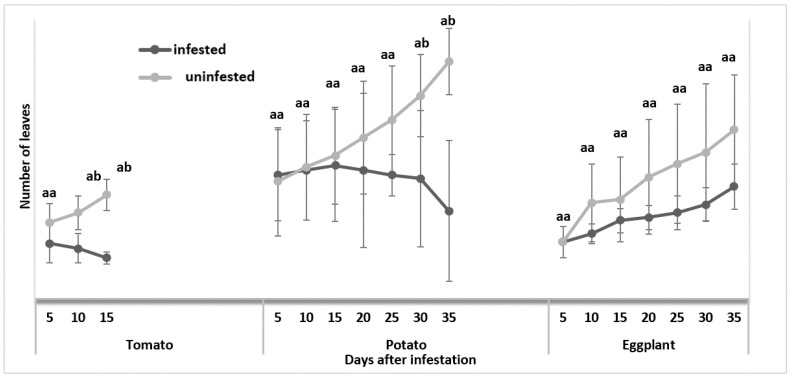
Comparison of the mean number of leaves found on uninfested and *Tuta absoluta*-infested plants. Means followed by different letters in the same bar column are significantly different at *p* < 0.05.

For the tomatoes, the infested host plants had a mean of 5 leaves while the uninfested plants had a mean of 13 leaves, with a *p*-value of <0.001, indicating a significant difference between the infested and uninfested host plants after 15 days of infestation (Figure 3).

**Figure 4:**
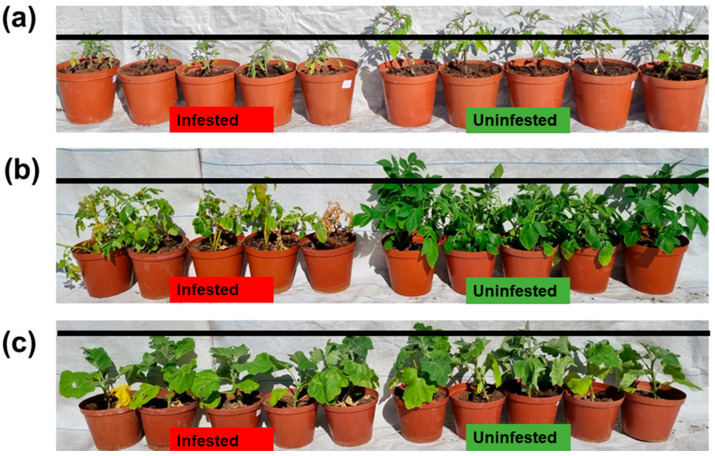
Comparisons of plant height, leaf color, and number of leaves between the uninfested and *Tuta absoluta*-infested tomato (**a**), potato (**b**), and eggplant (**c**).

For potatoes, there was a minimal difference between the infested and uninfested potato leaves during the early days of infestation (Figure 3). This could be attributed to less feeding damage caused by the L1 instar larvae, with damage becoming significant as the larvae grew and developed. Consequently, the leaves yellowed, and the foliage on infested plants deteriorated when compared to the uninfested plants (Figure 4), resulting in the mean number of leaves for the infested plants being 11, whereas the mean number of leaves was 29 for the uninfested plants, with a *p*-value of 0.003 after 35 days of infestation (Figure 3). During the course of the trial, some of the larvae successfully developed into adults.

For eggplant, the infested host plants had a mean number of leaves of 13 leaves, while the uninfested plants had a mean leaf number of 20, with a *p*-value of 0.08, indicating no significant difference between the infested and uninfested host plants after 35 days of infestation (Figure 3; also see Figure 4).

#### 3.1.2. Plant Height

**Figure 5:**
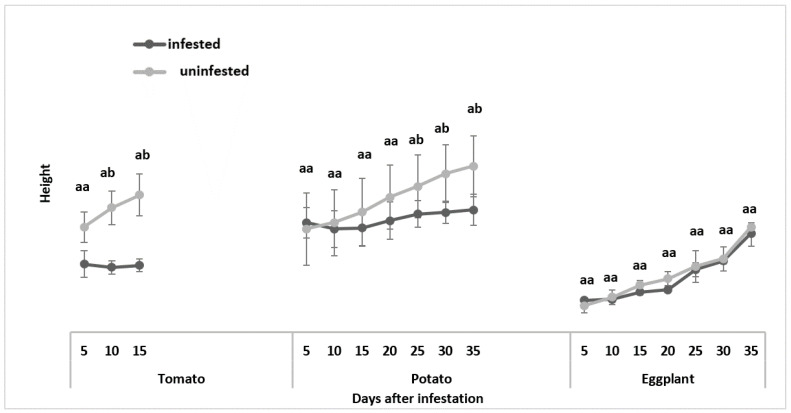
Comparison of mean plant height for uninfested and *Tuta absoluta*-infested plants. Means followed by different letters in the same bar column are significantly different at *p* < 0.05.

The feeding damage caused to the tomato by *T. absoluta* showed a drastic decline in the comparative growth rate of the plant, with the infested plants having a mean height of 12.6 cm, while the uninfested plants showed a mean plant height of 25.8 cm, with a *p*-value of <0.001, indicating a significant difference between the infested and uninfested host plants after 15 days of infestation. In addition, the infestation hindered the development of the plant’s main stem (Figure 5).

For potato, the infested plants had a mean height of 21.80 cm, while the uninfested host plants measured 31.20 cm, with a *p*-value of 0.008, indicating a significant difference between infested and uninfested host plants 35 days after infestation (Figure 5).

For eggplant, the infested plants had a mean height of 18.6 cm, while the uninfested eggplants had a mean height of 19.8 cm, with a *p*-value of 0.34, indicating no significant difference between the infested and uninfested host plants after 35 days infestation (Figure 5).

#### 3.1.3. Stem Diameter

**Figure 6:**
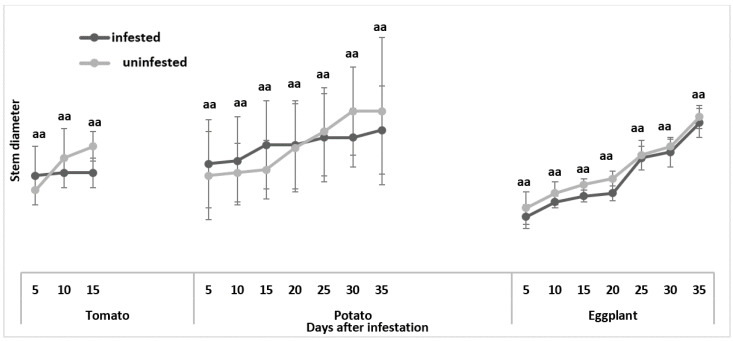
Comparison of mean stem diameter between uninfested and *Tuta absoluta*-infested plants. Means followed by different letters in the same bar column are significantly different at *p* < 0.05.

*Tuta absoluta* feeding damage had no significant effect on stem diameter across all three plant species (Figure 6).

#### 3.1.4. Fresh Weight, Dry Weight, and Loss of Moisture

The mean fresh weight of the infested tomato plants was 5.55 g, while the mean fresh weight of the uninfested tomato plants was 16.66 g, with a *p*-value of 0.004. Infested potato plants had a mean fresh weight of 24.02 g, while the uninfested plants had a mean fresh weight of 58.84 g, with a *p*-value of 0.004. The fresh weight of the infested eggplants was 42.78 g, while the fresh weight of the uninfested eggplants was 49.16 g, with a *p*-value of 0.35.

The infested tomato plants had a mean dry weight of 2.52 g, while their uninfested counterparts had a mean dry weight of 3.91 g, with a *p*-value of 0.07. The infested potato plants had a mean dry weight of 3.22 g, whereas the uninfested plants had a mean dry weight of 9.55 g, and a *p*-value of 0.002. The infested eggplant plants had a mean dry weight of 11.62 g, and their uninfested counterparts had a mean dry weight of 14.91 g, with a *p*-value of 0.21.

The difference between the fresh weight and dry weight of the infested tomato plants was 3.02 g, while the uninfested tomato plants had a mean weight of 12.44 g, with a *p*-value of 0.006. The difference between the fresh and dry weight for the infested potato plants was 20.8 g, whereas the uninfested potato plants had a mean weight of 41.24 g, with a *p*-value of 0.06. The difference between the fresh and dry weight for the infested eggplants was 31.154 g, while the uninfested eggplants had a mean weight of 34.24 g, with a *p*-value of 0.18.

#### 3.1.5. Potato Yields

**Figure 7:**
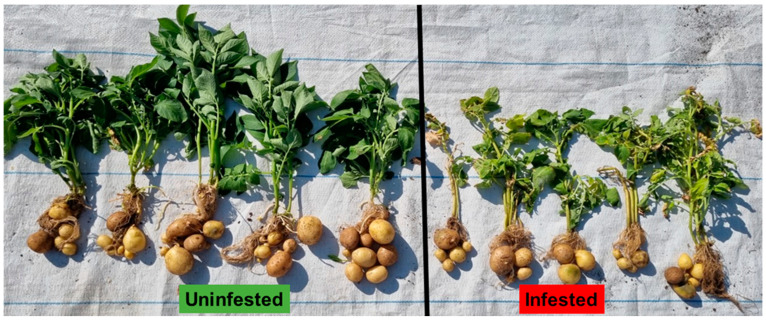
Yield comparison between the uninfested and *Tuta absoluta*-infested potato plants.

The potato tubers from the infested host plants were visually smaller compared to the potato tubers from the uninfested host plants (Figure 7). The potato tubers from the infested host plants had a mean weight of 69.68 g, while the potato tubers from the uninfested plants had a mean weight of 165.14 g, with a *p*-value of 0.05.

### 3.2. Host Plants Physiological Parameters

#### 3.2.1. Chlorophyll Content

**Figure 8:**
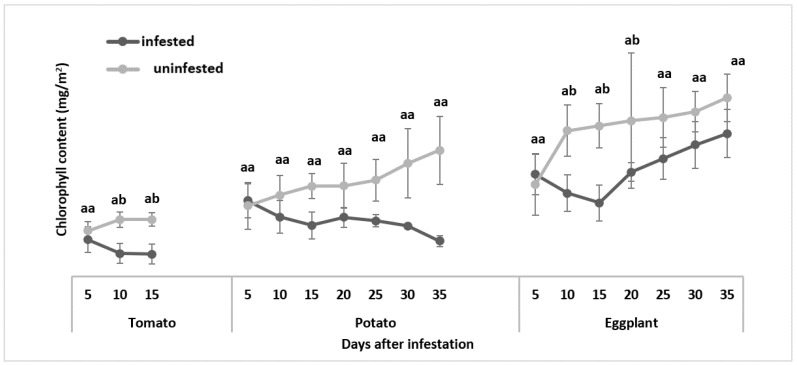
Comparison of mean chlorophyll content between the uninfested and *Tuta absoluta*-infested plants. Means followed by different letters in the same bar column are significantly different at *p* < 0.05.

The feeding damage caused by *T. absoluta* significantly reduced the chlorophyll content produced by the leaves of all three plant species (Figure 8). *Tuta absoluta* induced the yellowing, through to browning, of the leaves (Figure 4 and Figure 7) due to lesions caused by the pest. The more lesions created by the pest, the more chlorotic the host plants became. These were indications that the leaves were stressed. The chlorosis appeared to be very severe on tomato plants, followed by potato plants and then the eggplant plants. For the tomato plants, the infested host plants had a mean chlorophyll content of 3.9 mg/m^2^, while the uninfested plants had a mean chlorophyll content of 10.36 mg/m^2^, and a *p*-value of <0.001. For potatoes, the infested host plants had a mean chlorophyll content of 5.32 mg/m^2^, while the uninfested plants had a mean chlorophyll content of 22.32 mg/m^2^, and a *p*-value of 0.001. For eggplant, the infested host plants had a mean chlorophyll content of 25.3 mg/m^2^, while the uninfested plants had 31.6 mg/m^2^, with a *p*-value of 0.04, indicating a significant difference between the infested and uninfested host plants.

#### 3.2.2. Photosynthesis Rate

The mean photosynthetic rate for the infested tomato plants was 14.14 µmol CO_2_ m^−2^ s^−1^, while for the uninfested plants, it was 15.97 µmol CO_2_ m^−2^ s^−1^. This also applied to the potato host plants, for which the infested plants had a mean photosynthetic rate of 15.79 µmol CO_2_ m^−2^ s^−1^, while the uninfested plants had an amount of 16.90 µmol CO_2_ m^−2^ s^−1^. For eggplant, the infested plants had an amount of 17.14 µmol CO_2_ m^−2^ s^−1^, while the uninfested had an amount of 17.65 µmol CO_2_ m^−2^ s^−1^. When the statistical *t*-test was conducted, a significant difference was not detected, although there was clear damage to the leaves (Figure 4).

#### 3.2.3. Conductance

Conductance appeared to be similar between the infested plants and the uninfested plants, regarding tomato and potato. No significant difference was obtained for tomato and potato water conductance. For the tomato host plants, the mean conductance was −0.005 mol H_2_O m^−2^ s ^−1^ for the infested plants and −0.004 mol H_2_O m^−2^ s ^−1^ for the uninfested plants. For potato, the infested plants had a conductance of 0.011 mol H_2_O m^−2^ s ^−1^, while the uninfested plants had a conductance of 0. 014 mol H_2_O m^−2^ s ^−1^. For eggplant, a significant difference was obtained with a *p*-value of 0.05. The mean conductance for infested eggplant plants was −0.0001 mol H_2_O m^−2^ s ^−1^, while the uninfested plants had a conductance mean of 0.030 mol H_2_O m^−2^ s ^−1^.

#### 3.2.4. Transpiration Rate

*Tuta absoluta* feeds by mining a plant’s leaves, which leads to a papery appearance, indicating water loss. For tomato and eggplant, a significant difference was obtained between the infested and uninfested host plants. For potato, no significant difference was obtained between the infested and uninfested host plants. The transpiration rate for the infested tomato appeared to be higher than that for the uninfested plants. For tomato, the infested plants had a mean transpiration rate of −0.14 mmol m^−2^ s^−1^, while the uninfested plants had a mean of −0.10 mmol m^−2^ s^−1^, with a *p*-value of 0.01. For the potato plants, the infested plants had a mean of −2999.81 mmol m^−2^ s^−1^, and the uninfested plants had a mean of −1526.45 mmol m^−2^ s^−1^. For eggplant, the infested plants had a mean of 0.0032 mmol m^−2^ s^−1^, while the uninfested plants had a transpiration rate of 0.4908 mmol m^−2^ s^−1^, with a significant difference obtained regarding a *p*-value of 0.05.

#### 3.2.5. Intercellular Carbon Dioxide Concentration

The values between the infested and uninfested plants varied greatly in all the crops. The rate at which carbon dioxide enters the infested tomato plants appeared to be lower than the carbon dioxide entering the uninfested tomato plants. This entails that a lower amount of carbon dioxide enters the infested host plants. The damage to the leaves might have disrupted the flow of carbon dioxide. The infested tomato plants had a mean intercellular carbon dioxide concentration of 1825.52 μmol mol^−1^, while the uninfested plants had a mean of 3215.28 μmol mol^−1^. For potato, the intercellular carbon dioxide concentration for the infested plants was 0.2743 μmol mol^−1^, while the uninfested plants showed 0.3629 μmol mol^−1^. For eggplant, a mean intercellular carbon dioxide concentration of −8836.65 μmol mol^−1^ was detected, while the uninfested plants had an amount of 208.58 μmol mol^−1^, with a *p*-value of 0.05.

### 3.3. Correlation of Parameters

The correlation analysis results indicated that, for tomato, the leaves per plant and plant height, stem diameter and photosynthesis rate, chlorophyll content and photosynthesis rate, and transpiration rate and water conductance were positively correlated (Table 1). For potato, the chlorophyll content and photosynthesis rate and the transpiration rate and water conductance were positively correlated (Table 2). For eggplant, the leaves per plant and plant height, chlorophyll content and photosynthesis rate, and transpiration and water conductance were positively correlated, while the height and transpiration and the height and water conductance were negatively correlated (Table 3).

## 4. Discussion

The interaction between herbivores and host plants often leads to the impaired growth and capability of the latter [19]. *Tuta absoluta* fed on all three plant species under investigation, but the effects of its feeding damage on plant growth and physiological function were more pronounced on tomato and potato plants than on eggplant plants.

The infested leaves of tomato and potato appeared to be unhealthy, wilting, chlorotic, and necrotic. Neves et al. [20] explain that unhealthy leaves lead to a lower intake of the photosynthesis active flux, which is triggered by the strain and destruction of the photosynthesis system. The discoloration of the leaves before the plants could reach maturity suggested that there was a disturbance to plants’ physiological parameters. This is also supported by Pincebourde and Ngao [21]. Four physiological parameters, namely photosynthesis rate, transpiration rate, water conductance, and intercellular CO_2_ concentration, were investigated in the current study.

The tomato plants had completely defoliated, and the leaves had a brownish discoloration at termination (15 days after infestation). This also occurred for the potato plants, although the visible browning and defoliation were evident 35 days after infestation. This explains why the experiment was terminated at 15 days for the tomato plants and after 35 days for the other crops. This observation is similar to the study of Soliman and Imam [22], who found that *T. absoluta* leads to the destruction of the host plants. 

The biomass of the tomato-infested host plants was greatly affected, followed by that of potato, while eggplant was the least affected. For eggplant, the biomass was not affected; hence, eggplant recovered from the damage inflicted by *T. absoluta* and developed more leaves. Eggplant has demonstrated the ability to recover after plant stress [23]. The loss in biomass for infested host plants was due to the loss of moisture in the infested host plants [24]. Observing the dry mass, the infested host plants had a lower dry mass compared to the uninfested host plants. Insect infestations lead to low dry mass [20]. This also implicates that an attack by herbivores leads to a decrease in the assimilation rate of the host plants [19].

The height of the infested host was affected by *T. absoluta*. The destructive pest affected the plant’s ability to develop. Insect damage affects the growth, development, and life span of the plants [25]. The height remained stagnant, with the leaves damaged and the plant biomass significantly reduced in the infested host plants compared to the uninfested plants. This observation is similar to that of Cely et al. [17].

The results indicated that tomato plants infested with *T. absoluta* deteriorate quicker than potato and eggplant. A plant’s deterioration before maturity leads to reduced or a complete loss of yield. This became evident in the case of the tomato plants, where there was a 100% loss to the host plants 15 days after infestation.

In our investigation, *T. absoluta* was exposed to a “no-choice” test, where they could not move and/or feed on alternative host plants. The results indicated that in the absence of tomato, *T. absoluta* is highly likely to enact significant damage on potato fields, especially if there is a higher density of *T. absoluta*. This is likely to occur and hence the high reproduction of the destructive pest [26]. Pereyra and Sánchez [16] stated that under favorable conditions, *T. absoluta* could become a pest of great economic importance for potatoes–this study confirms that *T. absoluta* was given a no-choice test and was fed on potato, with this leading to the destruction of some potato host plants. In our investigation, we could not establish if *T. absoluta* could directly feed on the potato tubers; however, the infested potatoes’ host plants produced smaller and fewer tubers compared to the uninfested plants. This confirms the statements made by Desneux et al. [5] that *T. absoluta* can cause reduced yield in potatoes. *T. absoluta* can be found under the soil in a cocoon when they pupate; however, no symptoms of damage were observed on the tubers by the pupa.

Our study is similar to that found in several works of literature, namely that tomato plants are severely favored by *T. absoluta,* when compared to the other host plants [9,16,27] and this implicates that the genetic makeup of tomato makes it more susceptible to the pest. The leaf odor on tomato leaves makes it more suitable for *T. absoluta* [9]. It was also observed that the damage intensity caused by the pest was not inflicted at the same time as this, as the tomato damage was evident earlier (10 days after infestation), whereas it took longer regarding potato. Tomato plants could not defend themselves against the attack and deteriorated 15 days after infestation. In the case of potato, the damage was initially small, and became severe as the instar stages developed–hence the leaves progressively deteriorated towards termination 35 days after infestation.

The infestation by *T. absoluta* indicated a difference in values of leaf gas exchange parameters between the infested and uninfested plants. The leaf-mining by *T. absoluta* greatly affected the chlorophyll content of the infested host plants. When *T. absoluta* infested the plants, it led to the complete removal of its leaves, meaning that the chloroplast in the leaves was also removed, including the palisade layer of the leaf parenchyma tissue; hence, the reduction in chlorophyll content. The complete removal of the plant tissue from the leaves led to a change in photosynthesis activity. There was no significant difference in the photosynthesis rate between the infested and uninfested host plants five days after infestation. However, there was a significant reduction in, and significant difference between, the infested and uninfested host plant’s content, which was measured every five days. Each time the measurements of chlorophyll were recorded, a reduction was observed. With every measurement, the leaves of the infested plants, especially tomato and potato plants, were observed to be chlorotic, which became an indication of a reduction in the chlorophyll content.

Furthermore, chlorophyll content is regarded as a great indicator of the health status of a plant, as it is directly related to photosynthesis [28] and hence the high Pearson correlation coefficients between the photosynthesis rate and chlorophyll content from our correlation analysis findings (Table 1, Table 2 and Table 3). The photosynthetic rate lowered in all the infested plants. However, this reduction in the photosynthesis rate was not statistically significant. Therefore, the reduction in photosynthesis in all the infested host plants indicated that the destructive pest does affect photosynthesis in the plants. This is similar to the work conducted by Hussain et al. [29], in which it was found that insect damage may affect the photosynthetic rate. The reduction of photosynthesis in potato due to *T. absoluta* was also observed by Sperdouli [30]. The impact of reduced photosynthesis was observed during harvest, where the potato tubers and the yield of the infested potato were visibly reduced when compared to the uninfested potato host plants (Figure 7). When part of the plant tissue is removed due to insect feeding, the remaining plant tissue overcompensates, and this leads to an increase in the rate of photosynthesis [31], which is probably why the photosynthesis rate was not reduced significantly regarding statistical analysis.

Damage to the leaves leads to lower carbon dioxide intake (into the leaves) [20]. In our investigation, the intercellular carbon dioxide concentration appeared to be lower in all the infested host plants compared to the uninfested host plants, suggesting that damage inflicted upon the leaves by this destructive pest leads to a lower intake of carbon dioxide.

When the conductance of a plant is low, it causes a low amount of water and nutrients to pass through the stomata [32]; therefore, when plants are attacked by these pests, the health of the plant is in jeopardy, hence the limited water and nutrients. In our study, the conductance in the infested plants was lower than in the uninfested plants. This was not what we had expected because damaged leaves lead to higher conductance [20]. However, the herbivory mode of *T. absoluta*, of burrowing mines and causing a papery appearance of the leaf, probably causes different plant responses than just the chewing of the leaf. Superficial mines due to damage by *T. absoluta* possibly led to limited water flow within the plant.

Leaves per plant and plant height, chlorophyll content and photosynthesis rate, and transpiration rate and water conductance were constant and positively correlated in almost all three crops. The correlation between leaves per plant and plant height demonstrated that the taller the plants, the more leaves per plant produced, and the shorter the plant, the fewer the leaves produced. The positive correlation between leaves per plant and plant height is similar to results obtained by Wali and Kabura [33] and Onyia et al. [34]. No correlation was found between leaves per plant and plant height for potato. These results are not consistent with the ones found by Sattar et al. [35]. However, this could be because the results by Sattar et al. [35] were obtained from healthy host plants.

There is a close relationship between conductance and transpiration [20]. This observation was also consistent with our investigation. Transpiration and water conductance as a positive correlation in our investigation is similar to the results obtained by Acatrinei [36]. Acatrinei [36] also explained that this correlation is facilitated by leaf dehydration. A decrease in chlorophyll content becomes an indication that there is a decrease in the rate of photosynthesis, hence the high positive correlation between the chlorophyll content and photosynthesis rate. A positive correlation between the chlorophyll content and photosynthesis rate was also found by Buntin et al. [37]. For eggplant, we observed a positive correlation between plant height and photosynthesis rate. Eggplant was least damaged by *T. absoluta,* and the number of leaves increased significantly, maybe due to the correlation between the two parameters. A positive correlation between plant height and photosynthesis was also obtained by Yu et al. [38]. An increase in photosynthesis can lead to increased plant growth [39]. Eggplant has thick trichomes on the surface of its leaves [40], and these trichomes contribute to the boundary layer of the leaves [41]. A thick boundary layer leads to a low transpiration rate [41], which may explain the positive correlation for eggplant between plant height, transpiration, plant height, and conductance. Positive correlations for leaf gas exchange were also consistent with those obtained by Hamani et al. [42] and Ibrahim et al. [43].

To the best of our knowledge, no correlation studies have been conducted on growth parameters and leaf gas exchange in crops infested with *T. absoluta*. Therefore, a comparison of the correlation between growth parameters and leaf gas exchange was deduced from studies conducted on tomato, potato, and eggplant, and other similar studies on growth and gas exchange parameters when plants were under other stresses (instead of *T. absoluta* infestation).

*Tuta absoluta* has a major effect on the production of several host plants in South Africa and worldwide. Climatic conditions favorable to tomato and other Solanaceae crops are also the same favorable conditions for the survival and reproduction of *T. absoluta.* Therefore, there is a high likelihood of the long-time survival of these destructive pests [44], especially since most trusted strategies, such as chemicals to control the pest, are currently ineffective, which is the biggest problem faced by farmers and researchers.

Previous studies demonstrated that *T. absoluta* could develop and grow on tomato, potato, and eggplant. In this study, we documented the morphological and physiological damage inflicted by this pest on different host plants, detailing the reduction in plant biomass, development, disturbance of gas exchange, and reductions in yield. Although *T. absoluta* has been shown to successfully develop on tomato, potato, and eggplant, it has been reported to exhibit a higher oviposition preference and offspring fitness for tomato, followed by potato, then eggplant [45]. This higher preference for oviposition on tomato and potato has been explained in terms of the volatile organic compounds that deter oviposition on eggplant and the presence of higher amounts of terpenes in those plant species compared to eggplant that serve as oviposition stimulants [46]. In addition, the lower suitability of eggplant for infestation by *T. absoluta* has been explained in terms of the presence of 77 secondary metabolites that increase in abundance following infestations that are believed to deter feeding by *T. absoluta* [47]. Thus, the lower feeding damage by *T. absoluta* on eggplant is due to antixenosis (defined as the non-preference of insect pests for plants that lack stimuli for feeding or oviposition) and antibiosis (defined as adverse physiological responses experienced by the insect pest following the ingestion of plant material) resistance. We acknowledge the various areas the present study has not addressed, such as the level of economic injury caused by the pest on the crop, to formulate recommendations for farmers. It may therefore be necessary to emulate the study of Ghaderi et al. [48].

## 5. Conclusions

The results indicated that host plants responded negatively to the presence of the *T. absoluta*, considering the morphological and physiological parameters studied. The damage of the *T. absoluta* led to a disturbance in the number of leaves and the biomass produced by the plants and a reduction in the yield and assimilation rate due to the damage inflicted by interrupted plant gas exchange, nutrients, and water movements. The tomato was mostly affected and deteriorated much quicker, followed by the potato and eggplant. Although *T. absoluta* was not observed to inflict damage directly on the tubers, the infested host plants produced lower yields and smaller tubers compared to the uninfested potato host plants.

The feeding damage caused by *T. absoluta* affected the infested host plants’ growth and development (physiological function) differentially. Effects were higher on tomato, followed by potato, and then eggplant. Except for eggplant, the feeding damage slowed the development of leaves in the infested host plants. The height of the infested host plants remained stagnant, while the height of the uninfested host plants increased. This ultimately caused the lower fresh and dry weights of the infested host plant. *Tuta absoluta* instigated 100% loss to the tomato-infested host plants; hence, all the infested tomato host plants senescence and scotched 15 days after the initial infestation. The potato tubers of infested host plants had a lower quantity compared to the uninfested potato host plants. In all three host plants, the infested plants had a higher ratio of damaged leaves compared to the uninfested plants. The infested host plants had senescence and scorched leaves. As the plants grew, the number of leaves decreased in the infested host plants, while they increased in the uninfested host plants. We admit that these results are preliminary, and further extensive research is necessary to fully answer the question of host preference with an additional number of experimental plants and not only the three we included.

## Figures and Tables

**Table 1:** Pearson correlation coefficient (upper value) and *p*-value (lower value) for the tomato-infested host plant.

Tomato	LPP	PH	SD	CC	P	T_r_	C	Ci
LPP	-							
PH	0.93 0.02	-						
SD	0.81 0.09	0.61 0.27	-					
CC	0.52 0.36	−0.05 0.93	0.69 0.20	-				
P	0.85 0.06	0.43 0.47	0.85 0.07	0.84 0.07	-			
T_r_	−0.13 0.83	−0.38 0.52	−0.01 0.99	0.14 0.81	0.07 0.91	-		
C	−0.13 0.83	−0.38 0.52	0.00 0.99	0.15 0.81	0.08 0.91	1.00 0.006	-	
C_i_	0.05 0.93	0.35 0.56	−0.26 0.67	−0.26 0.67	−0.02 0.96	−0.73 0.10	−0.74 0.10	-

Note: LPP = Leaves per Plant; SD = Stem Diameter; CC = Chlorophyll Content; P = Photosynthesis Rate; Tr = Transpiration Rate; C = Conductance; Ci = Intercellular Carbon Dioxide Concentration.

**Table 2:** Pearson correlation coefficient (upper value) and *p*-value (lower value) for the potato-infested host plant.

Potato	LPP	SD	PH	CC	P	T_r_	C	Ci
LPP	-							
SD	−0.15 0.84	-						
PH	0.15 0.84	0.62 0.37	-					
CC	0.68 0.31	0.45 0.54	0.81 0.18	-				
P	−0.78 0.21	0.40 0.59	−0.33 0.66	0.74 0.05	-			
T_r_	0.69 0.30	0.48 0.51	0.20 0.66	0.62 0.37	−0.16 0.84	-		
C	0.70 0.29	0.45 0.54	0.18 0.81	0.61 0.38	−0.17 0.83	1.00 0.004	-	
C_i_	0.01 0.98	−0.46 0.53	−0.95 0.40	−0.66 0.33	0.33 0.66	0.08 0.92	0.11 0.89	-

Note: LPP = Leaves per Plant; SD = Stem Diameter; CC = Chlorophyll Content; P = Photosynthesis Rate; Tr = Transpiration Rate; C = Conductance; Ci = Intercellular Carbon Dioxide Concentration.

**Table 3:** Pearson correlation coefficient (upper value) and *p*-value (lower value) for the eggplant infested host plant.

Eggplant	LPP	PH	SD	CC	P	T_r_	C	Ci
LPP	-							
PH	0.90 0.03	-						
SD	0.21 0.73	−0.13 0.83	-					
CC	0.69 0.19	−0.65 0.23	−0.10 0.86	-				
P	−0.70 0.19	0.89 0.04	0.28 0.64	0.70 0.04	-			
T_r_	0.79 0.11	−0.92 0.04	0.45 0.44	0.41 0.49	−0.73 0.15	-		
C	0.80 0.10	−0.92 0.02	0.44 0.45	0.41 0.49	−0.74 0.15	1.00 0.001	-	
C_i_	−0.14 0.82	0.40 0.50	−0.44 0.45	0.37 0.54	0.38 0.50	−0.67 0.21	−0.66 0.22	-

Note: LPP = Leaves per Plant; SD = Stem Diameter; CC = Chlorophyll Content; P = Photosynthesis Rate; Tr = Transpiration Rate; C = Conductance; Ci = Intercellular Carbon Dioxide Concentration.

## Data Availability

The dataset utilized in this study is available upon request.
